# Comparison of clinical and laboratory characteristics between severe and non-severe dengue in paediatrics

**DOI:** 10.1371/journal.pntd.0011839

**Published:** 2023-12-19

**Authors:** Nurul Liyana Idrus, Shamsuriani Md Jamal, Afliza Abu Bakar, Hashim Embong, Nurul Saadah Ahmad

**Affiliations:** 1 Department of Emergency Medicine, Faculty of Medicine, Universiti Kebangsaan Malaysia, Jalan Yaacob Latif, Bandar Tun Razak, Cheras, Kuala Lumpur, Malaysia; 2 Emergency and Trauma Department, Hospital Bukit Mertajam, Jalan Kulim, Bukit Mertajam, Pulau Pinang, Malaysia; Australian Red Cross Lifelood, AUSTRALIA

## Abstract

**Background:**

The timely identification of severe dengue in peadiatric patients is of utmost importance, as any delay in diagnosis could lead to an irreversible state of shock potentially leading to fatal consequences. The primary aim of our study was to characterize dengue severity in paediatric patients based on initial symptoms, signs, and laboratory investigation of their presentation in the emergency department.

**Methodology:**

We conducted a retrospective data retrieval from the medical records of 254 paediatric patients who had been diagnosed with confirmed cases of dengue fever. The clinical characteristics were compared between severe and non-severe dengue. Multiple logistic regression analysis was utilised to elucidate the variables that exhibited associations with severe dengue.

**Results:**

A total of 254 paediatric patients were included, among whom 15.4% (n = 39) were diagnosed with severe dengue. Multiple logistic regression analysis identified lethargy, systolic blood pressure (SBP) below 90 mmHg, capillary refilled time (CRT) longer than 2 seconds, ascites, and hepatomegaly were independently associated with severe dengue.

**Conclusion:**

In paediatric patients, severe dengue is associated with specific clinical indicators, including lethargy, low systolic blood pressure, prolonged capillary refill time (CRT), and the presence of ascites and hepatomegaly. Identifying these clinical features early is crucial for primary care physicians, as it enables accurate diagnosis and timely intervention to manage severe dengue effectively.

## Author summary

Cheras is a suburban area located in the Klang Valley region of Malaysia. In recent years, the population of Cheras has grown rapidly due to urbanization and economic development in the surrounding areas. Cheras has a relatively high population density (N = 135,823, 8,489 / km^2^) with a mix of high-rise apartment buildings, landed residential properties, and commercial areas. The area is well-connected to other parts of the Klang Valley via major highways and public transportation, which also contributes to the population density. The present study documented clinical and laboratory profiles of 254 paediatric patients with dengue who presented in the emergency department between November 2018 to November 2019 at the only hospital in Cheras, a prevalent dengue area. Patients had an average age of 13 years old, and the male-to-female ratio was nearly balanced. More than 15% had severe dengue manifestation. We found the independent clinical risk factors for severe dengue in paediatric patients include lethargy, systolic blood pressure (SBP) below 90 mmHg, capillary refilled time (CRT) longer than 2 seconds, ascites, and hepatomegaly. Early recognition of these manifestations will assist primary care physicians in making correct diagnosis with timely intervention and management. The presence of dengue in a high-density population and rapidly growing zone warrants further holistic actions to reduce its impact on children’s health.

## 1.0 Introduction

Dengue virus (DENV), an arbovirus transmitted by Aedes mosquitoes, remains a significant public health concern, especially in tropical and subtropical regions, where severe cases can result in high mortality rates. According to data from the World Health Organization (WHO), modelling estimates suggest there are approximately 390 million dengue infections each year, of which 96 million results in clinical manifestations. Moreover, a staggering 70% of the global dengue burden is concentrated in the Asia region [1]. Over the past two decades, the reported number of cases has increased over eightfold, surging from over 500,000 cases in the year 2000 to 5.2 million cases in 2019 [2].

In Malaysia, the situation regarding dengue is indeed alarming. The number of dengue cases and the incidence rate have increased significantly from 7,103 cases in 2000 to 130,101 cases in 2019 [3]. This substantial rise in dengue cases emphasises the pressing need for effective prevention and control measures to mitigate the impact of this mosquito-borne disease in the country.

Previously, dengue haemorrhagic fever was predominantly a disease that primarily affected paediatric patients [4]. It has been estimated by WHO, that approximately 500,000 patients with severe dengue require hospitalization each year, with a substantial portion of these being paediatric patients[5]. Surveillance data from various Asian countries have consistently shown that infants under 1 year of age and paediatric patients aged 4 to 9 years old, face the highest risk of severe dengue disease [6]. In Malaysia, the annual percentage of cases among paediatric patients has increased by approximately 20% from the year 2014 to 2019 [3].

The management of severe dengue in paediatric patients presents a complex challenge, starting with the difficulty of diagnosing dengue fever itself. This challenge arises for several reasons. Dengue infection is inherently dynamic, displaying a wide clinical spectrum, and often compounded with other febrile illnesses. During both febrile and critical phases, clinical manifestations and laboratory parameters can overlap, making diagnosis and management challenging. Moreover, several common tropical illnesses can mimic dengue fever-like illness (DFLI) such as chikungunya, leptospirosis, influenza and more recently coronavirus-19, further complicating diagnosis [7,8].

In paediatric patients, dengue often leads to profound vascular leakage and rapid shock but haemorrhagic manifestation is less common compared to adults [9]. In a study by Wakimoto and colleagues in 2015, several factors associated with the severity of dengue in paediatric patients were identified. These factors included hepatomegaly, lethargy, bleeding, abdominal pain, haemoconcentration, and thrombocytopenia [10]. The study emphasized that understanding the specific characteristics of dengue infection in paediatric patients is crucial for the early detection of severe dengue and for initiating appropriate treatment in such cases.

In Malaysia, there is a significant lack of comprehensive data regarding the clinical characteristics of dengue infection in paediatric patients. Given the rising incidence of dengue infections in Malaysia, there is an urgent need to thoroughly examine the clinical profile of this infection among the paediatric population. The primary objective of this study was to identify the risk factors associated with the severity of dengue in paediatric patients who visited the emergency department of a tertiary university hospital.

## 2.0 Methods

### Ethics statement

All data was extracted from the existing hospital database and analysed anonymously to protect patient privacy. This study was approved by the Research and Ethical Committee of the UKMMC with a designated research code FF-2020-394.

### 2.1 Study design

Between November 2018 and November 2019, we conducted a cross-sectional retrospective study by reviewing the medical records of paediatric patients diagnosed with dengue fever at the emergency department (ED) of the Universiti Kebangsaan Malaysia Medical Centre (UKMMC). UKMMC is an urban tertiary teaching hospital located in Kuala Lumpur, Malaysia.

### 2.2 Case selection

The study included cases based on the following inclusion criteria: 1) Confirmation of dengue through either a positive dengue serology test, detection of dengue-specific NS-1 antigen using a dengue ELISA kit, or identification of dengue immunoglobulin M and G (IgM, IgG) antibodies in the acute phase serum using enzyme-linked immunosorbent assay (ELISA), and 2) individuals aged 19 years or younger. Meanwhile, the subsequent cases were excluded: 1) Patients with concurrent infections, 2) Individuals who were transferred to other medical facilities, and 3) Patients who left the hospital against medical advice.

### 2.3 Study protocol and data collection

Initially, we conducted an online search to gather information on dengue patients who had been reported to the Vector Borne Disease Control Division of the Ministry of Health in Malaysia. This search encompassed details about patients, their addresses, and whether they were probable or confirmed dengue cases, as reported by the hospital. Subsequently, we identified the patients’ registration hospital numbers from the hospital information system and procured their medical records. The following data were acquired: 1) Sociodemographic information, 2) Clinical characteristics, encompassing findings from physical examinations and laboratory results, and 3) Patient disposition. All the data collected were coded to ensure confidentiality.

Dengue cases were categorised into severe and non-severe dengue. Severe dengue was defined according to WHO guidelines, by the presence of any of the following features: 1) evidence of plasma leakage that could lead to shock (referred to as dengue shock syndrome) with or without fluid accumulation, 2) significant bleeding 3) altered level of consciousness such as coma, and convulsions, 4) severe gastrointestinal involvement such as jaundice, and 5) severe organ impairment including acute liver failure, acute renal failure, encephalopathy, cardiomyopathy [4]. Dengue patients who did not exhibit these features were categorized as non-severe dengue, even if they presented with symptoms such as abdominal pain, vomiting, lethargy, and diarrhea (common warning signs in dengue), and were hemodynamically stable.

### 2.4 Statistical analysis

Statistical analysis was performed using Statistical Package for the Social Science Software (SPSS, Chicago, IL, USA). Categorical variables were expressed in frequency and proportion. Comparisons of clinical manifestations across the severity of dengue infections were analysed using Pearson Chi-Square tests and Fisher’s exact test. The normally distributed continuous variables were reported in means with standard deviation (SD) and non-normally distributed continuous variables were reported in median with interquartile range. Comparisons of laboratory parameters across the severity of dengue infection were analysed using independent-samples t-test or Mann-Whitney U test. Univariate analysis was performed first to check the significant association for each risk factor (variables). These significant variables were selected for multivariable logistic analysis using the backward likelihood ratio (LR) method. For numerical variables, the linearity in the logit for each variable was checked. Selected variables were checked for interactions and multicollinearity. Any variables that had a P value < 0.05 were considered strong independent risk factors for severe dengue.

## 3.0. Results

Out of the total number of dengue patients retrieved, which amounted to 16,076, only 1.58% constituted paediatric patients. In this subset, the study comprised 254 cases, and within this, 39 patients (15.3%) were categorized as severe dengue cases. The sociodemographic characteristics of the selected cases are detailed in Table 1. Among the patients, nearly two-thirds (58.3%, n = 148) were male with a median age of 13 years old (IQR 8–16 years old). Notably, there were no significant differences observed in the sociodemographic characteristics between those with severe dengue and those with non-severe dengue. Most of the patients, totaling 80% (n = 204), were admitted to the hospital, while 16.1% (n = 41) were admitted to the critical care unit.

**Table 1:** Sociodemographic characteristics of dengue in paediatric patients (N = 254).

Demographic data	Frequency N = 254	Non-severe N = 215	Severe dengue N = 39	P-value	
Age, median (IQR)	13 (8–16)	13 (8–16)	14 (11–17)	0.206	
Gender, N(%)				
Male	148 (58.3)	127 (59.1)	21 (53.8)	0.543	
Female	106 (41.7)	88 (40.9)	18 (46.2)		
Race, N (%)					
Malay	192 (75.6)	162 (75.3)	30 (76.9)	0.101	
Chinese	43 (16.9)	40 (18.6)	3 (7.7)		
Indian	10 (3.9)	7 (3.3)	3 (7.7)		
Others	9 (3.5)	6 (2.8)	3 (7.7)		
Time presentation to ED from onset of symptoms (days), mean ± SD	4.50 ± 1.48	4.56 ± 1.45	4.15 ± 1.61	0.116	
Locality					
Within Klang Valley Outside Klang Valley	250 (98.4) 4 (1.6)	212 (98.6) 3 (1.4)	38 (97.4) 1 (2.6)	0.590	
Disposition					
Discharge Admit to general ward. Admit to critical care unit	50 (19.7) 163 (64.2) 41 (16.1)	50 (23.3) 154 (71.6) 11 (5.1)	0 (0) 9 (23.1) 30 (76.9)	0.001*	

Data is expressed as n (%), median (range) and mean ±SD.

P-value determined by χ-square test, Fischer’s Exact test, Mann-Whitney U test and independent samples t test where appropriate.

*Values are significant at P-value<0.05

Table 2 provides an overview of the clinical characteristics observed in paediatric patients. Notably, there were statistically significant differences in several clinical features between the non-severe and severe dengue groups. All patients (100%) presented with fever. Among severe dengue patients, lethargy was observed in 92.3% of cases, while abdominal pain was reported in 64.1%. However, bleeding from multiple sources was found in only 20.9% of severe dengue cases. Additionally, significant differences were noted in the presence of clinical indicators, including lethargy, rashes, prolonged CRT (>2 seconds), hepatomegaly, liver tenderness, pleural effusion, ascites and systolic blood pressure when comparing non-severe and severe dengue cases.

**Table 2:** Clinical manifestations among paediatric patients in non-severe and severe dengue (N = 254).

Clinical manifestations	Overall cases N = 254	Non-severe dengue N = 215	Severe dengue N = 39	P-value
Fever	254 (100)	215 (100)	39	-
Lethargy	174 (68.5)	138 (64.2)	36 (92.3)	0.001*
Vomiting	99 (39.0)	79 (36.7)	20 (51.3)	0.087
Diarrhea	78 (30.7)	65 (30.2)	13 (33.3)	0.699
Abdominal pain	136 (53.5)	111 (51.6)	25 (64.1)	0.151
Rashes	65 (25.6)	62 (28.8)	3 (7.7)	0.005*
Epistaxis	15 (5.9)	13 (6)	2 (5.1)	0.823
Bleeding evidence	53 (20.9)	43(20.0)	10 (25.6)	0.425
CRT>2sec	17 (6.7)	02 (0.9)	15 (38.5)	0.001*
Hepatomegaly	41 (16.1)	28 (13)	13 (33.3)	0.002*
Liver tenderness	19 (7.5)	11 (5.1)	8 (20.5)	0.001*
Pleural effusion	24 (9.4)	12 (5.6)	12 (30.8)	0.001*
Ascites Systolic blood pressure mmHg	7 (2.8) 95 (90–102)	1 (0.5) 96 (90–103)	6 (15.4) 88 (81–94)	0.001* 0.001*

Data is expressed as n (%), median (range), and mean ±SD.

P-value determined by χ-square test, Fischer’s Exact test, Mann-Whitney U test, and independent samples t test where appropriate.

*Values are statistically significant at P-value <0.05.

Concerning the laboratory parameters outlined in Table 3, statistically significant differences (p<0.05) were observed in hemoglobin, platelet count, and lactate levels between the non-severe and severe dengue groups. Specifically, severe dengue patients exhibited lower serum hemoglobin levels, much lower platelet count, and higher lactate levels compared to non-severe dengue patients.

**Table 3:** Laboratory parameters among paediatric patients in non-severe and severe dengue (N = 254).

	No. of cases	Overall cases	Non-severe dengue	Severe dengue	P-value
Hemoglobin (g/dL)	N = 254	12.6 ± 1.5	12.8 ± 1.4	11.9 ± 1.8	0.002*
Leucocyte (x100^3^/mL)	N = 254	2.4 (1.9–3.5)	2.5 (1.9–3.5)	2.4 (1.5–3.2)	0.348
Platelet (x100^3^/mL)	N = 254	90.36 ± 57.44	96.98 ± 57.86	53.13 ± 37.42	0.001*
Hematocrit (g/dL)	N = 254	42.97 ± 4.65	42.79 ± 4.42	44.07 ± 5.76	0.192
Alanine Transaminase (U/L)	N = 229	40.0 (21.5–100.5)	38.00 (21.25–92.00)	55.00 (23.50–169.50)	0.102
Aspartate Transaminase (U/L)	N = 19	131.0 (65.0–348.0)	265.00 (65.25–478.50)	105.00 (64.00–241.00)	0.253
Lactate (mmol/L)	N = 175	1.85 ± 0.83	1.70 ± 0.65	2.43 ± 1.13	0.001*

Data is expressed as median (range) and mean ±SD.

P-value determined by χ-square test, Fischer’s Exact test, Mann-Whitney U test, and independent samples t test where appropriate.

*Values are statistically significant at P-value <0.05

The results of single logistic regression analysis for the independent variables which include age, day of illness upon presentation to the emergency department, lethargy, vomiting, abdominal pain, rashes, systolic blood pressure, prolonged CRT (> 2 seconds), pleural effusion, ascites, hepatomegaly, liver tenderness, hemoglobin level, hematocrit, platelet, alanine transaminase, aspartate transaminase and lactate indicated significant difference with a minimum threshold of 0.25.

The overall predictive ability of the Multivariable Logistic Regression model for severe dengue in paediatric patients was quite high, at 93.3%. This indicates a strong ability of the model to predict severe dengue cases. Additionally, the model displayed an excellent receiver operating characteristic (ROC) curve with an area under the curve (AUC) of 0.936, further supporting its predictive accuracy.

Notably, in the Multivariable Linear Regression (MLR) analysis, several factors were identified as independently associated with severe dengue among paediatric patients. These factors included lethargy (b = 8.16, p = 0.01), systolic blood pressure (b = 17.14, p = 0.01), prolong CRT exceeding 2 seconds (b = 15.39, p = 0.009), ascites (b = 27.28, p = 0.008), and hepatomegaly (b = 5.2, p = 0.006). These findings suggest that these factors can serve as significant predictors of severe dengue in paediatric patients, as demonstrated in Table 4.

**Table 4:** Factors associated with severe dengue (using Multiple Logistic Regression–Backward Method).

Variables	Adjusted OR	(95% Cl OR)	X^2^ stat. (df)	P values
Lethargy Yes No	8.16 1.00	(1.57; 42.29)	6.25 (1)	0.01*
Abdominal pain Yes No	0.38 1.00	(0.13; 1.16)	2.87 (1)	0.09
Rashes Yes No	0.28 1.00	(0.06; 1.27)	2.73 (1)	0.10
Systolic blood pressure (SBP)				
<90mmHg	17.14	(2.48;118.66)	11.26 (3)	0.01*
91-95mmHg	12.68	(1.77; 90.95)	8.28 (1)	0.004
96-102mmHg	2.68	(0.38; 18.93)	6.38 (1)	0.012
>103mmHg	1.00	-	0.97 (1)	0.32
Capillary refill time > 2 seconds Yes No	15.39 1.00	(2.00; 118.45)	6.89 (1	0.009*
Ascites Yes No	27.28 1.00	(2.35; 317.33)	6.96 (1)	0.008*
Hepatomegaly Yes No	5.20 1.00	(1.61; 16.83)	7.58 (1)	0.006*

Data is expressed as Adjusted Odds Ratio (OR) and 95% Confidence Interval (CI)

* Values are statistically significant at P- value < 0.05

### 4.0 Discussion

The number of dengue cases and the incidence rate in Malaysia has increased dramatically since the year 2014. The highest number of reported cases was recorded in 2019 with an incidence rate reaching 390.4 cases per 100,000 population [11]. This increase highlights a notable public health concern and underscores the importance of dengue prevention and control efforts in the region. This study revealed that in the year 2019, the prevalence of dengue infection among paediatric patients was 1.58% with the median age of paediatric patients experiencing severe dengue being 14 years old. This study is consistent with previous studies conducted by Pothapregada et al 2016 and Faridi et al 2008, both of which also demonstrated that older paediatric patients aged more than 6 years old were more frequently affected by dengue infection [12,13]. Likewise, a study conducted in Malaysia by Ahmad et al in 2018 also showed that the majority of dengue patients included in their research were aged more than 15 years old [14]. This aligns with the trend observed in our study and further emphasizes the prevalence of dengue infection among older individuals in the region. Recently, in a systematic review conducted by Tshten et al. in 2021, it was highlighted that there has been a noticeable shift in the incidence of dengue infection toward older paediatric patients [15]. Despite this shift, severe dengue remains a significant cause of morbidity and mortality in paediatric patients [16]. This emphasizes the continued importance of addressing and managing dengue infection among older paediatric patients.

Malaysia has implemented a revised classification system from WHO that categorizes dengue into non-severe and severe dengue [4]. Non-severe dengue is further divided into dengue with warning signs and dengue without warning signs. The clinical manifestations of dengue infections can vary from mild to life-threatening and are typically classified into three phases: febrile phase, critical phase, and recovery phase [11]. Primary care physicians play a crucial role in recognising the clinical manifestations of severe dengue and providing timely intervention to prevent fatal outcomes. Their vigilance and prompt action are vital in managing dengue cases effectively and ensuring the well-being of patients.

In our study, we have identified that the primary predictors of severe dengue are lethargy, SBP below 90 mmHg, prolonged CRT (> 2 seconds), ascites, and hepatomegaly. These findings affirm the significance of these factors as warning signs, consistent with the revised classification of dengue [4]. We discovered that paediatric patients who presented with lethargy were eight times more likely to develop severe dengue compared to those without lethargy. These findings align with the results of the previous study, highlighting the consistent association between lethargy and increased risk of severe dengue in paediatric patients [10]. Bedside assessment of circulation, encompassing factors like colour, CRT, peripheries temperature, pulse volume, and rate (CCTV-R), offers an initial impression of a dengue patient’s overall perfusion and hemodynamic status. In our research, we observed that patients with prolonged CRT exceeding 2 seconds were 15 times more likely to develop severe dengue. Capillary refill time, being a non-invasive and easily performed parameter serves as a valuable surrogate marker for assessing hypoperfusion and hemodynamic instability. Consequently, vigilant monitoring and judicious fluid management for these patients are of paramount importance to prevent fatal outcomes.

Low systolic blood pressure, along with the presence of pleural effusion and ascites, serve as important indicators of plasma leakage in paediatric dengue patients. We identified SBP of less than 90 mmHg as an independent factor, with paediatric patients in this category being 17 times more likely to develop severe dengue. Ascites; another independent risk factor was associated with 27 times greater likelihood of severe dengue development. These findings are consistent with prior research, including a systematic review, which demonstrated the clinical importance and the significance of signs indicating plasma leakage in dengue infection [17–19]. A study in Thailand by Pongpan et al in 2013, also linked SBP below 90mmHg to severe dengue [20]. Pleural effusions and ascites are considered warning signs of fluid accumulation in dengue [11]. These clinical findings result from increased capillary permeability in dengue infection, causing fluid to accumulate in the potential spaces such as the pleural and peritoneal cavities. The ongoing vascular permeability and plasma leakage can lead to hypovolaemia and shock, ultimately culminating in dengue shock syndrome (DSS). Early detection of these clinical signs is crucial for timely intervention. Failure to recognise these signs can lead to serious complications.

In our review, we observed that both hepatomegaly and liver tenderness were statistically significant clinical variables. Specifically, we found a positive association between hepatomegaly and severe dengue, with paediatric patients presenting with hepatomegaly being five times more likely to develop severe dengue. These findings are consistent with similar associations demonstrated in other studies. Pongpan et al in 2013 (AOR:43.44, P<0.001), Wichman et al in 2004 (AOR: 7.5, P = 0.02), and Mena Lora et al in 2014 (AOR: 2.60, P: not described) have also reported similar relationships between hepatomegaly and severe dengue [20–22]. Hepatic involvement in dengue infection can manifest as hepatomegaly, tender liver, or elevated serum transaminase level. While we found that a tender liver was statistically significant, it was not identified as an independent risk factor in our study. Unfortunately, we couldn’t establish a significant association between liver enzyme transaminases and severe dengue, primarily because not all dengue-positive patients underwent serum liver enzyme investigations. Hepatomegaly (defined as liver enlargement greater than 2cm) and liver tenderness are included in the warning signs outlined in the WHO guidelines. Their presence may indicate transient acute hepatitis in dengue. Liver involvement is frequently observed in paediatric patients with dengue infection, although fulminant acute liver failure is rare [11]. Overall, our study contributes valuable insights into the clinical manifestations associated with severe dengue among paediatric patients in an endemic area.

### Limitations

This study was conducted as a retrospective, cross-sectional study with convenience sampling, and it was carried out in a single-centre tertiary hospital. It is important to note that the findings of this study may not be representative of the general population. Although this study had a retrospective design, it can serve as an initial step in generating hypotheses that warrant further exploration through larger prospective studies in Malaysia. Throughout this study, some data values were missing. However, efforts were made to minimise these issues by conducting a thorough analysis of clinical information in the medical records, which had been documented by physicians and nursing staff. Additionally, the laboratory parameters were recorded in a computerized system at the hospital (UKMMC), which helped reduce the occurrence of missing values in this specific data category.

## 5.0 Conclusion

Lethargy, systolic blood pressure below 90 mmHg, capillary refill time exceeding 2 seconds, ascites, and hepatomegaly have been identified as independent clinical risk factors associated with severe dengue in paediatric patients. The early recognition of these clinical manifestations is of utmost importance as it enables primary care physicians to make accurate diagnoses and initiate timely interventions and management strategies. This proactive approach can significantly improve patient outcomes and reduce the severity and complications of dengue infection among paediatric population.

## References

[1] Brady OJ, Gething PW, Bhatt S, Messina JP, Brownstein JS, Hoen AG, et al. Refining the Global Spatial Limits of Dengue Virus Transmission by Evidence-Based Consensus. PLoS Negl Trop Dis. 2012;6. doi: 10.1371/journal.pntd.0001760 22880140 PMC3413714

[2] Bhatt S, Gething PW, Brady OJ, Messina JP, Farlow AW, Moyes CL, et al. The global distribution and burden of dengue. Nature. 2013;496: 504–507. doi: 10.1038/nature12060 23563266 PMC3651993

[3] MOH. Kematian Demam Denggi Tahun 2021 & 2020. Minist Heal Malaysia. 2021.

[4] WHO. DENGUE Guidelines for diagnosis, Treatment, Prevention and control. World Heal Organ. 2009. 23762963

[5] WHO. Dengue, Dengue Haemorrhagic Fever and Dengue Shock Syndrome in the Context of the Integrated Management of Childhood Illness Department of Child and Adolescent Health and Development. Discuss Pap child Heal. 2005.

[6] Huy R, Buchy P, Conan A, Ngan C, Ong S, Ali R, et al. Surveillance nationale du dengue au Cambodge 1980–2008: Tendances épidémiologiques et virologiques, et impact du contrôle des vecteurs. Bull World Health Organ. 2010;88: 650–657. doi: 10.2471/BLT.09.073908 20865069 PMC2930366

[7] Leelarasamee A, Chupaprawan C, Chenchittikul M, Udompanthurat S. Etiologies of acute undifferentiated febrile illness in Thailand. J Med Assoc Thail. 2004;87. 15222513

[8] Nacher M, Douine M, Gaillet M, Flamand C, Rousset D, Rousseau C, et al. Simultaneous dengue and COVID-19 epidemics: Difficult days ahead? PLoS Negl Trop Dis. 2020;14: 1–8. doi: 10.1371/journal.pntd.0008426 32797035 PMC7428060

[9] Simmons Cameron P, Farrar Jeremy J, van Vinh Chau Nguyen BW. Dengue. N Engl J Med. 2012;366: 1423–1432. doi: 10.1056/NEJMra1110265 22494122

[10] Wakimoto MD, Camacho LAB, Guaraldo L, Damasceno LS, Brasil P. Dengue in children: A systematic review of clinical and laboratory factors associated with severity. Expert Rev Anti Infect Ther. 2015;13: 1441–1456. doi: 10.1586/14787210.2015.1100534 26536064

[11] Ministry of Health Malaysia. Clinical Practice Guidelines 2020 on Management of Dengue in Children. Ministry of Health Malaysia. 2020. Available: http://www.moh.gov.my

[12] Pothapregada S, Kamalakannan B, Thulasingham M, Sampath S. Clinically profiling paediatric patients with dengue. J Glob Infect Dis. 2016;8: 115–120. doi: 10.4103/0974-777X.188596 27621562 PMC4997795

[13] Faridi MMA, Aggarwal A, Kumar M, Sarafrazul A. Clinical and biochemical profile of dengue haemorrhagic fever in children in Delhi. Trop Doct. 2008;38: 28–30. doi: 10.1258/td.2007.006158 18302860

[14] Ahmad MH, Ibrahim MI, Mohamed Z, Ismail N, Abdullah MA, Shueb RH, et al. The sensitivity, specificity and accuracy of warning signs in predicting severe dengue, the severe dengue prevalence and its associated factors. Int J Environ Res Public Health. 2018;15. doi: 10.3390/ijerph15092018 30223572 PMC6163319

[15] Tsheten T, Clements ACA, Gray DJ, Adhikary RK, Wangdi K. Clinical features and outcomes of COVID-19 and dengue co-infection: a systematic review. BMC Infect Dis. 2021;21: 1–9. doi: 10.1186/s12879-021-06409-9 34340682 PMC8327042

[16] Ooi E, Gubler DJ. Dengue in Southeast Asia: Epidemiological characteristics and strategic challenges in disease prevention. Cad Saude Publica. 2009;25: 115–124. doi: 10.1590/s0102-311x2009001300011 19287856

[17] Rodrigo C, Sigera C, Fernando D, Rajapakse S. Plasma leakage in dengue: a systematic review of prospective observational studies. BMC Infect Dis. 2021;21: 1–11. doi: 10.1186/s12879-021-06793-2 34670495 PMC8527656

[18] Nelwan EJ. Early Detection of Plasma Leakage in Dengue Hemorrhagic Fever. Acta Med Indones. 2018;50: 183–184. 30333266

[19] Srikiatkhachorn A. Plasma Leakage in Dengue Hemorrhagic Fever Anon. Thromb Haemost. 2017;102: 1042–1049. doi: 10.1160/TH09-03-0208 Plasma 19967133 PMC5527705

[20] Pongpan S, Wisitwong A, Tawichasri C, Patumanond J, Namwongprom S, Casimir GJ, et al. Clinical Study Development of Dengue Infection Severity Score. ISRN Pediatr. 2013;845876. Available: 10.1155/2013/845876 24324896 PMC3845515

[21] Wichmann O, Hongsiriwon S, Bowonwatanuwong C, Chotivanich K, Sukthana Y, Pukrittayakamee S. Risk factors and clinical features associated with severe dengue infection in adults and children during the 2001 epidemic in Chonburi, Thailand. Trop Med Int Heal. 2004;9: 1022–1029. doi: 10.1111/j.1365-3156.2004.01295.x 15361117

[22] Mena Lora AJ, Fernandez J, Morales A, Soto Y, Feris-Iglesias J, Brito MO. Disease severity and mortality caused by dengue in a Dominican paediatric population. Am J Trop Med Hyg. 2014;90: 169–172. doi: 10.4269/ajtmh.13-0440 24218410 PMC3886416

